# Prolidase could be considered a sign of inflammation associated with cigarette smoking

**DOI:** 10.3389/fmed.2024.1347688

**Published:** 2024-04-04

**Authors:** Berna Botan Yıldırım, Sevsen Kulaksızoglu

**Affiliations:** ^1^Department of Pulmonary Diseases, Faculty of Medicine, Baskent University, Konya, Türkiye; ^2^Department of Medical Biochemistry, Faculty of Medicine, Baskent University, Konya, Türkiye

**Keywords:** cigarette smoking, prolidase, total antioxidant status, total oxidant status, respiratory function parameters

## Abstract

**Objectives:**

Smoking causes inflammation, thickening, and narrowing of the airways. This inflammatory process is a reaction to free radicals and oxidants. Smoking affects collagen metabolism and tissue remodeling. Prolidase enzyme hydrolyzes iminodipeptides with hydroxyproline and C terminal proline. It plays a crucial role in the metabolism of collagen and the remodeling of the matrix. The present study aims to reveal the association of prolidase with inflammation caused by smoking and to compare serum prolidase levels with oxidative-antioxidative status in healthy individuals.

**Methods:**

A total of 76 participants (38 smokers and 38 nonsmokers) were involved in the present study. Serum cotinine levels were measured to show the exposure to nicotine in tobacco smoke by using the competitive inhibition enzyme immunoassay method. Serum prolidase, total oxidant status (TOS), and total antioxidant status (TAS) were determined by the enzyme-linked immunosorbent (ELISA) method, respectively. The correlation between smoking, serum prolidase levels, TOS, and TAS was investigated.

**Results:**

TAS and serum prolidase levels of smokers were considerably lower than those in non-smokers (*p* < 0.001, *p* = 0.012 respectively). However, no differences were observed in TOS between the two groups. There was no statistically significant correlation between serum prolidase levels, TAS, and TOS. Moreover, no relationship was observed between respiratory function parameters and serum prolidase levels.

**Conclusion:**

To the best of our knowledge, the present study is the first study to demonstrate the role of prolidase in smoking-related inflammation. The results achieved in the present study suggest that smoking creates an imbalance in the oxidant-antioxidant activity. Smoking decreases prolidase levels, leading to decreased collagen turnover. Chronic pulmonary disease might be related to this decrease in collagen turnover.

## Introduction

Smoking leads to 30% of all cancer deaths in developed countries (1). Lung cancer is the major malignancy caused by smoking (2). Smoking is also the most significant factor associated with chronic obstructive pulmonary disease (COPD) (3). Tobacco contains many free radicals and oxidants that damage airway epithelium, causing airway inflammation. Moreover, reactive oxygen species such as hydroxyl radicals, hydrogen peroxide, superoxide, and nitric oxide are produced during inflammation (4). DNA, proteins, carbohydrates, and lipids are oxidatively damaged by these reactive oxygen radicals (5). The cumulative effect of all oxidants is defined as TOS (6). Cells have developed antioxidant defense mechanisms to remove these harmful oxidative reactions. These mechanisms consist of enzymes such as catalase, superoxide dismutase, paraoxonase, and glutathione peroxidase and vitamins such as ascorbic acid, β-carotene, and tocopherols (7). TAS measures all these serum antioxidants (8). The ratio of TOS to TAS levels is indicated as the oxidative stress index (OSI).

Several studies suggested that cigarette smoking affects collagen metabolism and tissue remodeling (9, 10). It causes inflammation of the airways and then thickening of the walls due to scarring and remodeling. Further progression leads to the narrowing of the small airways. Therefore, tissue remodeling and controlled alterations of extracellular matrix (ECM), in which matrix metallopeptidases (MMPs) are involved, are necessary. Prolidase (EC. 3.4.13.9), a member of the MMP family, hydrolyzes the imidodipeptides at the C-terminal proline or hydroxyproline (11). It plays a very important role in collagen metabolism and matrix remodeling. Prolidase activity was analyzed in various diseases such as cardiac hypertrophy (12), type 2 diabetes mellitus (13), osteoarthritis (14), chronic liver disease (15), chronic uremia (16), and bronchial asthma (17). In addition to an imbalance in the oxidant-antioxidant status, prolidase might also be associated with inflammation caused by cigarette smoking. To date, serum prolidase levels in smokers have not been investigated adequately. In the present study, serum prolidase levels of smokers were compared to nonsmokers to elucidate its role in inflammation associated with cigarette smoking. The correlation between the oxidative-antioxidative status and serum prolidase levels in these patients was also examined.

## Materials and methods

### Patients

The present study involved 38 healthy smokers (19 male and 19 female) and 38 healthy nonsmokers (18 male and 20 female), who applied to the Check-up Polyclinic and the Department of Chest Disease at the Baskent University Konya Medical Hospital between 2021 and 2022. The smoking group was defined by using a questionnaire as those who were smokers at the time of the study and who had smoked at least 100 cigarettes (18). Smoking history was obtained as self-reported pack years calculated as cigarettes smoked per day/20 multiplied by years smoked. None of them were using electronic cigarette. Control subjects were also not passive smokers. Table 1 included demographic details such as age, sex, and body mass index (BMI). Exclusion criteria included those with hypertension, diabetes mellitus, coronary artery disease, chronic obstructive pulmonary disease, collagen tissue disorders, ischemia, and hemorrhage. None of the patients were taking any medication (especially antioxidant and anti-inflammatory drugs) and alcohol. This study complies with the ethical principles of the Declaration of Helsinki. Baskent University Medical School’s ethics committee approved this Project (Project no. KA21/390). Every patient signed an informed written consent document.

**Table 1:** Demographic characteristics of participants.

	Gender	*N*	Mean	Std. Deviation	*t*-test	Sig.
Age (years)	Female	38	28.86	7.282	−1.476	0.144
	Male	38	31.23	6.695		
BMI	Female	38	21.50	3.799	−2.550	0.013*
	Male	38	23.41	2.658		
Smoking (packages/year)	Female	19	6.500	3.157	−2.438	0.020*
	Male	19	10.97	7.347		

*p < 0.05 was considered statistically significant.

### Spirometric measurements

A digital spirometer was used to test the lung function (Quark PFT, Cosmed, Italy). Forced expiratory volume in 1 s (FEV_1_), forced vital capacity (FVC), peak expiratory flow (PEF), forced mid-expiratory flow (FEF25-75%), and FEV1/FVC were computed as stated by the American Thoracic Society and the European Respiratory Society (19).

### Laboratory measurements

Blood samples were centrifuged at 3000 rpm for 10 min. The obtained serum was kept at −80°C until analysis. Serum cotinine levels were analyzed to determine the degree of cigarette smoke exposure and the amount of nicotine ingested. Serum cotinine levels were measured by using the competitive inhibition enzyme immunoassay method with a USCN life science kit (Cat. No CET058Ge, Wuhan USCN Business Co. Ltd., PRC). The intra-assay precision, expressed as coefficient variation (CV), was <10%, whereas the inter-assay precision, expressed as CV, was <12%. The concentrations of serum cotinine were expressed as ng/ml. Serum prolidase levels were measured since enzyme activities are related to enzyme levels (20). Serum prolidase levels were quantified with a USCN ELISA kit for Peptidase D (Cat No SEM011Hu, Wuhan USCN Business Co. Ltd., PRC). The intra-assay CV was <10%, whereas the inter-assay CV was <12%. The concentrations of serum prolidase were given as ng/ml. The ELISA method was used to measure the levels of serum TAS and TOS, using Rel Assay Diagnostics kits, Turkey. The intra-assay CV was 3.3%. The inter-assay CV was 2.8%. Serum TOS levels were given as μmol H_2_O_2_/L. Serum TAS concentrations were given as mmol Trolox/L. The OSI was defined as the TOS to TAS percent ratio.

OSI = TOS/TAS = (μmol H_2_O_2_/L)/ (mmol Trolox/L).

### Statistical analysis

The statistical analysis was performed by using SPSS 15.0.0 statistical package software. All the variables were found to have normal distribution tested by using the Kolmogorov–Smirnov test. All results were expressed as the mean ± standard deviation. The factor effect (smoking) was analyzed on many parameters related to each other. Therefore, MANOVA was used to determine the effect of cigarette smoking on dependent measurements. *Post hoc* tests were not performed for groups because less than three groups exist (Table 2).

**Table 2:** MANOVA analysis with power.

Dependent Variable	Sum of Squares	df	Mean Square	*F*	Sig.	Noncent. Parameter	Observed Power^a^
Cotinine Contrast Error	1,137,537 799278.2	1 74	1137536.895 10801.057	105.317	0.000	105.317	1.000
Prolidase Contrast Error	1,047 0.469	1 74	1,047 0.006	165.257	0.000	165.257	1.000
OSI Contrast Error	750.699 568.530	1 74	750.699 7.683	97.711	0.000	97.11	1.000
TAS Contrast Error	6.240 68.840	1 74	6.240 0.930	6.708	0.012	6.708	0.724
TOS Contrast Error	63.983 5327.099	1 74	63.983 71.988	0.889	0.349	0.889	0.154

The *F* tests the effect of GROUP. This test is based on the linearly independent pairwise comparisons among the estimated marginal means.

a. Computed using alpha = 0.05.

Demographic variables were analyzed by using the independent sample t-test. Multivariate analyses were conducted for the effects of smoking because of correlated measurements. The correlation in values was evaluated by using Pearson’s correlation test. Statistical significance was set at *p* < 0.05.

Sample size calculations depend on mainly three variables (Prolidase, TOS, TAS). Previous data for these variables were obtained from the manuscript “Isbilen, E., Kulaksizoglu, S., et al., Role of prolidase activity and oxidative stress biomarkers in unexplained infertility, Int J Gynecol Obstet. 2022: 156; 430–435 (20). First published online: 4 September 2021.” Interquartile range, median, expected significant difference between the two groups, and power (95%) were used for sample size calculation. Sample sizes were calculated to be 38, 32, and 37 for Prolidase, TOS, and TAS, respectively. Consequently, the sample size was chosen to be 38 for each group. Therefore, a total of 76 participants were enrolled for smoking and non-smoking group parameter comparisons.

## Results

**Figure 1:**
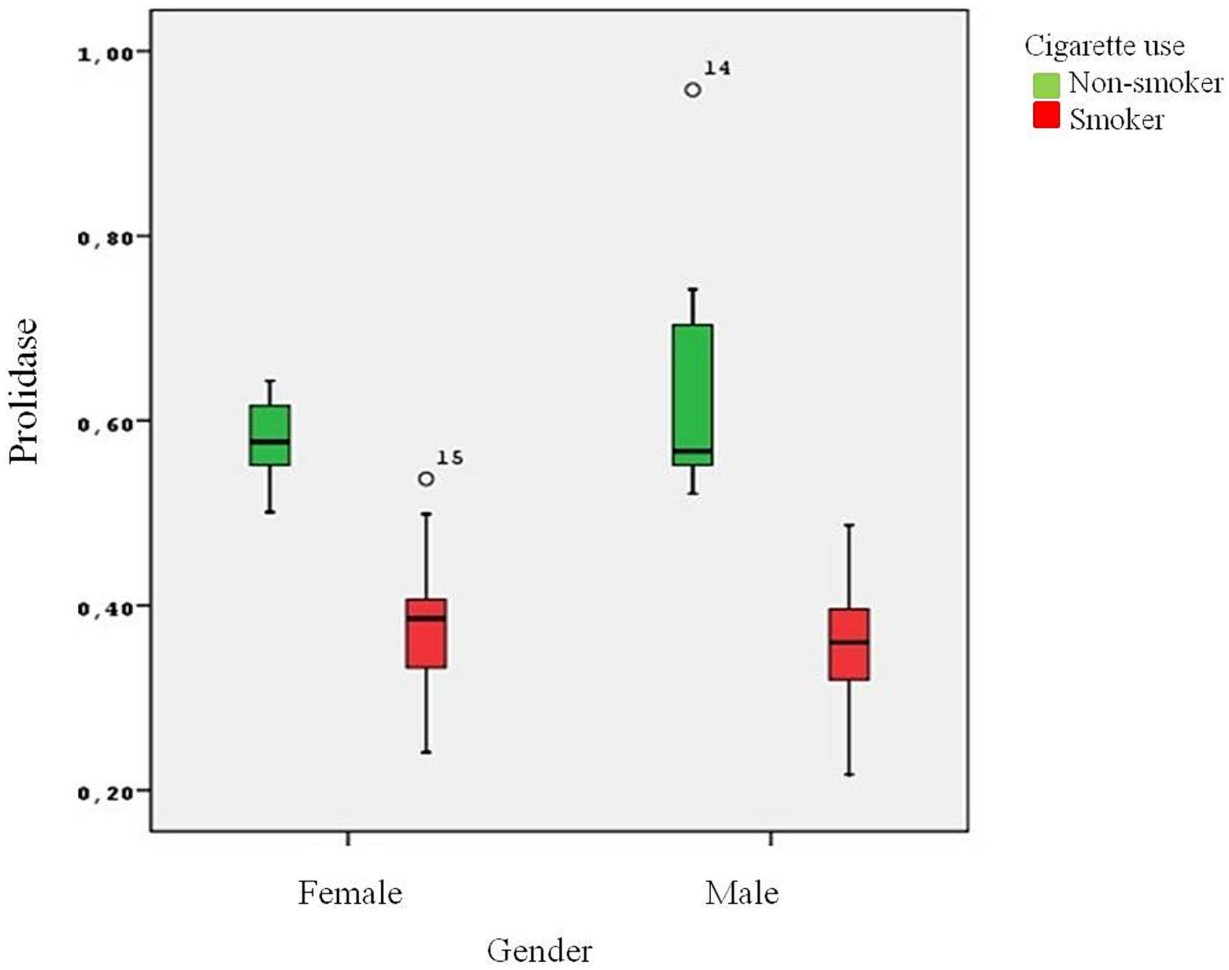
The effect of cigarette smoking on serum prolidase levels.

The present study involved 38 healthy smokers (19 male and 19 female) and 38 healthy nonsmokers (18 male and 20 female) with an average age of 30.05 ± 7.05 years. There was no statistically significant difference between the groups in terms of age. The male group had a higher BMI than the female group (*p* = 0.013). The mean number of packages smoked per year was 8.73 ± 6.02 packages/year. As expected, the male group smoked more packages on average than the female group (*p* = 0.02) (Table 1). Serum cotinine levels were analyzed to determine the degree of cigarette smoke exposure and the amount of nicotine ingested. Higher levels of cotinine were found in the smoker group in comparison to the nonsmokers (*p* < 0.001). Plasma prolidase level was significantly lower in the smoker group than in the control group (*p* < 0.001) (Figure 1).

Smokers were found to have considerably lower serum prolidase and TAS levels than non-smokers (*p* < 0.001, *p* = 0.012 respectively) (Table 3). However, no differences were determined in TOS levels between the two groups. OSI levels were significantly higher in the smoker group (*p* < 0.001).

**Table 3:** Serum Cotinine, Prolidase, TAS, TOS, and OSI levels of participants.

	Cigarette_use	N	Mean	Std. Deviation	Test	Sig.
Cotinine (ng/ml)	Non-smoker	38	146.0	50.06	*t* = 10.5317	<0.001*
	Smoker	38	390.6	138.18		
Prolidase (ng/ml)	Non-smoker	38	0.602	0.088	*t* = 12.855	<0.001*
	Smoker	38	0.367	0.070		
OSI%	Non-smoker	38	5.178	2.466	*t* = −9.885	<0.001*
	Smoker	38	11.463	3.046		
TAS (mmol Trolox/L)	Non-smoker	38	1.520	1.269	*t* = 2.590	0.012*
	Smoker	38	0.946	0.499		
TOS (μmol H_2_O_2_/L)/	Non-smoker	38	12.819	10.497	*t* = 0.943	0.349
	Smoker	38	10.984	5.812		

MANOVA: Pillais’s Trace (0.995), Wilks’ Lambda (0.005), Hotelling’s Trace (185.590), Roy’s Largest Root (185.590) tests are significant at level < 0.001.

**Figure 2:**
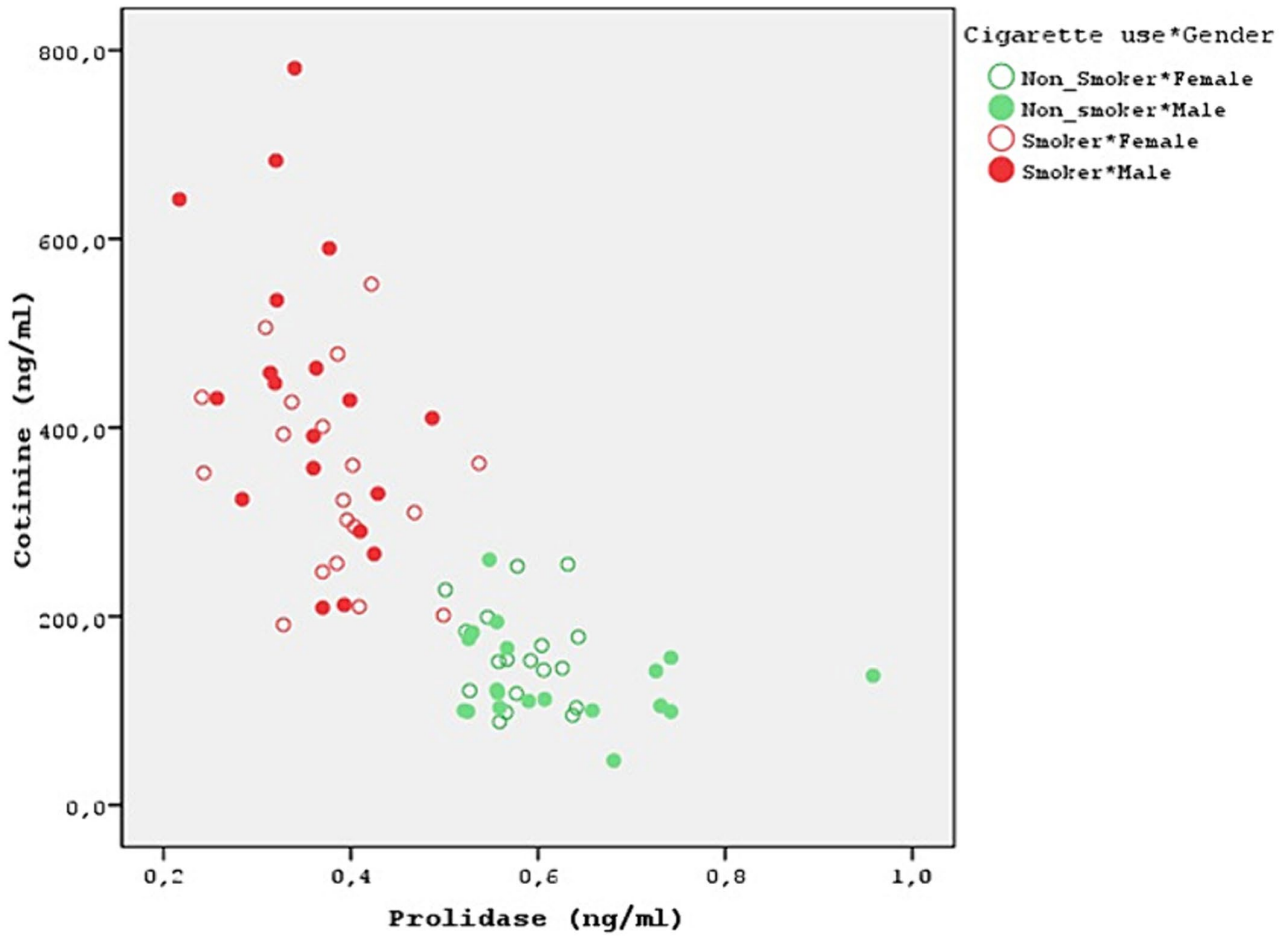
Relationship between prolidase and cotinine.

Both smokers and nonsmokers showed a positive correlation between serum prolidase and TAS levels. Moreover, serum prolidase and TOS levels were found to have a negative correlation. However, the difference was not statistically significant (Table 4). A statistically significant negative correlation was determined between prolidase and OSI levels (*r* = −0.629). This negative correlation was more evident in the male smokers (*r* = −0.601). Moreover, prolidase and cotinine levels also had a statistically significant and negative correlation (*r* = −0.739) (Figure 2). Even though this correlation was not statistically significant in nonsmokers, the smokers showed a significant negative correlation (*r* = −0.380). Furthermore, the negative correlation between prolidase and cotinine levels was higher in the male smokers when compared to the female smokers (*r* = −0.435).

**Table 4:** The correlation between prolidase and other parameters.

Correlation with Prolidase		All	Cigarette use		Non-smoker		Smoker	
			Nonsmoker	Smoker	Female	Male	Female	Male
Cotinine	PearsonCorr.	−0.739 (**)	**−0.239**	−0.380 (*)	**−0.168**	**−0.217**	**−0.260**	**−0.435**
	Sig. (2-tailed)	0.000	0.149	0.019	0.492	0.373	0.283	0.062
	N	76	38	38	19	19	19	19
TAS	PearsonCorr.	**0.210**	**−0.118**	**0.133**	**−0.084**	**−0.081**	**−0.120**	**0.408**
	Sig. (2-tailed)	0.069	0.481	0.427	0.732	0.742	0.626	0.083
	N	76	38	38	19	19	19	19
TOS	PearsonCorr.	**0.142**	**−0.109**	**−0.054**	**0.194**	**−0.274**	**−0.291**	**0.114**
	Sig. (2-tailed)	0.221	0.514	0.749	0.427	0.256	0.227	0.643
	N	76	38	38	19	19	19	19
OSI (%)	PearsonCorr.	−0.629 (**)	**0.083**	**−0.099**	**−0.172**	**0.108**	**0.214**	−0.601 (**)
	Sig. (2-tailed)	0.000	0.619	0.555	0.482	0.661	0.380	0.006
	N	76	38	38	19	19	19	19
FEV1 (%)	PearsonCorr.	**−0.012**	**0.245**	**−0.102**	**0.182**	**0.253**	**0.136**	**−0.291**
	Sig. (2-tailed)	0.915	0.138	0.543	0.456	0.295	0.579	0.226
	N	76	38	38	19	19	19	19
FVC (%)	PearsonCorr.	**−0.159**	**0.088**	**−0.244**	**−0.118**	**0.142**	**−0.248**	**−0.262**
	Sig. (2-tailed)	0.170	0.600	0.140	0.631	0.562	0.305	0.279
	N	76	38	38	19	19	19	19
FEV1/FVC	PearsonCorr.	−0.293 (*)	−0.361 (*)	−0.126	0.182	−0.546 (*)	−0.033	−0.234
	Sig. (2-tailed)	0.010	0.026	0.450	0.457	0.016	0.892	0.335
	N	76	38	38	19	19	19	19
PEF (%)	PearsonCorr.	**0.077**	**0.112**	**−0.109**	**−0.048**	**0.115**	**0.018**	**-,0.50**
	Sig. (2-tailed)	0.511	0.504	0.516	0.846	0.640	0.942	0.540
	N	76	38	38	19	19	19	19
MEF25_75_%	PearsonCorr.	**0.103**	**0.239**	**0.059**	**0.365**	**0.295**	**0.335**	**−0.041**
	Sig. (2-tailed)	0.376	0.148	0.724	0.124	0.220	0.161	0.869
	N	76	38	38	19	19	19	19

**Corr. is significant at the 0.01 level (2-tailed). * Corr. is significant at the 0.05 level (2-tailed).

No significant correlation could be found between prolidase levels and spirometric values FEV_1_, FVC, PEF, and FEF25-75%. However, the correlation between FEV1/FVC and prolidase levels was negative (*r* = −0.293, p00.01). This negative correlation was at a higher level in the nonsmokers than the smokers (*r* = −0.361). Particularly, it was more evident among the male nonsmokers (*r* = −0.549, *p* = 0.016) (Table 4).

## Discussion

There are 10^17^ oxidant molecules in every cigarette puff (21). Exposure to these oxidants in cigarette smoke causes airway epithelial injury. Numerous health issues, including cancer, cardiovascular disease, and pulmonary disorders, are influenced by this elevated oxidative stress (21). Ceylan et al. demonstrated that one of the main causes of COPD is the increase in oxidative stress (22). Gencer et al. detected high levels of plasma lipid peroxidation in COPD patients (23). Moreover, an imbalance in the oxidant-antioxidant activity leads to pathological processes affecting airways. As stated by Beck-Speier et al., free radical-mediated pathologies such as ischemia–reperfusion and asthma are often influenced by the imbalance between oxidative stress and antioxidant system (24). In the present study, TAS levels of smokers were lower than those of nonsmokers (*p* = 0.012). The smokers had significantly higher OSI levels (*p* < 0.002). The results achieved in the present study are consistent with those reported by Mahmood et al., who observed that TAS values in smokers were significantly lower than those in nonsmokers (25). Tavilani et al. observed that plasma TAS levels of patients with COPD and smokers were significantly lower than the nonsmoker group (4). This decrease in the total antioxidant capacity may be related to the increased oxidant production that consumes antioxidants. Gilks et al. reported that antioxidant enzyme levels may decrease severely during exposure to oxidants repeatedly (26). However, serum TOS levels did not differ between the smokers and the nonsmokers in the present study. In a study carried out by Aslan et al., the difference between smokers and nonsmokers in terms of serum TOS levels was not statistically significant (5). Zinellu et al. reported that the bronchoalveolar lavage fluid of COPD patients showed an increase in several oxidative stress biomarkers in contrast to healthy controls. On the other hand, the analysis of serum oxidant levels did not yield any difference (27). The higher concentrations of these oxidants in bronchoalveolar lavage fluid may be observed in the lungs because of direct exposure to oxidants of cigarette smoke and their short lifespan may not be sufficient to affect systemic levels, as shown in the present study.

Smoking also down-regulates MMPS concentration. Raitio et al. showed that smoking decreased the amount of MMP-9 in skin biopsy samples (28). Smokers have less hydroxyproline accumulation. Prolidase, a MMP family member, is essential for the remodeling of the ECM and the metabolism of collagen and proline recycling is crucial for collagen synthesis. Collagen is an important component of lung tissue and disruption of the collagen cycle can impair the structural integrity and function of the airways. Gencer et al. reported significantly reduced prolidase activity in COPD patients when compared to controls (23). In the study carried out by Ekin et al., prolidase activity decreased in patients with COPD (29). Ergin et al. showed that prolidase activity in patients with COVID-19 was statistically significantly lower when compared to healthy individuals (30). Uysal et al. demonstrated that prolidase activity might contribute to determining the severity of COPD (31). However, in a study carried out by Kaleli et al., prolidase activity was found to be at a higher level in bronchial asthma (17). Türkbeyler et al. detected enhanced prolidase activity in an experimental lung fibrosis model (32). As stated by Gumus et al., pulmonary tuberculosis was associated with increased serum prolidase activity and patients with cavitary disease had higher serum prolidase activity than patients with noncavitary disease (33). In the present study, significantly lower serum prolidase levels were found in the smokers (Figure 1). This decrease in prolidase levels suggests a potential alteration in collagen recycling and ECM remodeling processes in smokers, which may contribute to the pathogenesis of lung diseases, especially those associated with smoking.

The control group was formed by questioning the passive smoking status of the volunteers and blood cotinine level was studied to confirm this. None of the groups in this study was using electronic cigarette. A popular technique for substituting tobacco consumption is the electronic cigarette. Electronic cigarette smoke contains nicotine. It induces changes in oxidative stress and serum prolidase levels (34). Moreover, thirdhand smoke refers to the residual tobacco smoke contaminants that remain on surfaces and in dust after the cigarette has been extinguished. This can include substances such as nicotine, heavy metals, and various chemicals present in tobacco smoke. Even though the visible smoke is gone, these harmful substances can linger on surfaces such as clothing, furniture, walls, and carpets. Third-hand smoking was overlooked in the present study. After the small number of cases, this is one of the biggest limitations of the present study. It’s important to acknowledge the limitations of this study, such as its cross-sectional design and the potential influence of unmeasured confounders. Future research should explore the longitudinal effects of thirdhand smoking on oxidative stress markers and prolidase activity. Cotinine levels were at the upper limit of normality in all cases and were statistically significant in smokers (*p* < 0.001) (Table 3). Moreover, a strong negative relationship was discovered between prolidase and cotinine levels (*r* = −0.739). This negative correlation is more pronounced in male smokers, suggesting a potential gender difference in the effect of smoking on collagen metabolism and oxidative stress (*r* = −0.435) (Figure 2). This also suggests that prolidase levels and oxidative stress parameters change even in the case of passive smoking and thirdhand smoking. In the present study, both smokers and nonsmokers showed negative correlations between TOS levels and prolidase levels and positive correlations between TAS levels. However, these correlations were not statistically significant.

Even though no significant correlation was found between spirometric values (FEV1, FVC, PEF, FEF25-75%) and prolidase levels, there was a poor and negative correlation between FEV1/FVC ratio and prolidase levels, particularly in nonsmokers. This suggests that changes in collagen turnover, as reflected by changes in prolidase activity, may affect airway function. Further studies with larger sample sizes are necessary to elucidate the relationship between prolidase levels and lung function parameters.

Despite several studies in recent years, there are many conflicting data on prolidase levels in diseases. To date, serum prolidase levels have not been investigated adequately in smokers. The present study compared serum prolidase levels between smokers and nonsmokers to elucidate its role in inflammation associated with smoking. It was aimed to show whether serum prolidase levels can be regarded as a marker of inflammation in cigarette smoking and to determine the relationship between prolidase and oxidative-antioxidative status. The results achieved in this study indicate that cigarette smoking decreased serum prolidase levels. This may be interpreted as decreased collagen turnover. Smoking led to an imbalance in the activity of antioxidants and oxidants. However, there was no statistically significant correlation between serum prolidase levels, TAS, and TOS. Further studies with larger groups may be useful to clarify the effects of cigarette smoking.

## Data availability statement

The raw data supporting the conclusions of this article will be made available by the authors, without undue reservation.

## Ethics statement

The studies involving humans were approved by the Ethics Committee of Baskent University Medical School (Project no. KA21/390). The studies were conducted in accordance with the local legislation and institutional requirements. The participants provided their written informed consent to participate in this study. Written informed consent was obtained from the individual(s) for the publication of any potentially identifiable images or data included in this article.

## Author contributions

BY: Investigation, Writing – original draft, Writing – review & editing. SK: Validation, Writing – original draft, Writing – review & editing.

## References

[1] IARC Working Group on the Evaluation of Carcinogenic Risks to Humans. Tobacco smoke and involuntary smoking. IARC Monogr Eval Carcinog Risks Hum. (2004) 83:1–1438. PMID: 15285078 PMC4781536

[2] Hecht SS DeMarini DM. Tobacco smoke and its constituents In: Baan RA Stewart BW Straif K, editors. Tumour site concordance and mechanisms of carcinogenesis. Lyon: International Agency for Research on Cancer (2019) 33979073

[3] Mannino DM Buist AS. Global burden of COPD: risk factors, prevalance and future trends. Lancet. (2007) 370:765–73. doi: 10.1016/S0140-6736(07)61380-4 17765526

[4] Tavilani H Nadi E Karimi J Goodarzi MT. Oxidative stress in COPD patients, smokers and non-smokers. Respir Care. (2012) 57:2090–4. doi: 10.4187/respcare.01809 22710284

[5] Aslan R Ruhusen K Civi S Tasyurek E. The correlation of the total antioxidant status (TAS), total oxidant status (TOS) and paraoxonase activity (PON1) with smoking. Clin Biochem. (2014) 47:393–7. doi: 10.1016/j.clinbiochem.2013.10.002, PMID: 24440837

[6] Erel O. A new automated colorimetric method for measuring total oxidant status. Clin Biochem. (2005) 38:1103–11. doi: 10.1016/j.clinbiochem.2005.08.008, PMID: 16214125

[7] Zinellu E Zinellu A Fois AG Carru C Pirina P. Circulating biomarkers of oxidative stres in chronic obstructive pulmonary disease: a systematic review. Respir Res. (2016) 17:150. doi: 10.1186/s12931-016-0471-z, PMID: 27842552 PMC5109807

[8] Erel O. A novel automated direct measurement method for total antioxidant capacity using a new generation, more stable ABTS radical cation. Clin Biochem. (2004) 37:277–85. doi: 10.1016/j.clinbiochem.2003.11.015, PMID: 15003729

[9] Wong LS Martins-Green M. Firsthand cigarette smoke alters fibroblast migration and survival: implications for impaired healing. Wound Repair Regen. (2004) 12:471–84. doi: 10.1111/j.1067-1927.2004.12403.x, PMID: 15260813

[10] Nakamura Y Romberger DJ Tate L Ertl RF Kawamoto M Adachi Y. Cigarette smoke inhibits lung fibroblast proliferation and chemotaxis. Am J Respir Crit Care Med. (1995) 151:1497–503. doi: 10.1164/ajrccm.151.5.7735606, PMID: 7735606

[11] Piotr W Elzbieta W Weiss MS. Prolidase-a protein with many faces. Biochimie. (2021) 183:3–12. doi: 10.1016/j.biochi.2020.09.017 33045291

[12] Demirbag R Yıldız A Gur M Yilmaz R Elçi K Aksoy N. Serum prolidase activity in patients with hypertension and its relation with left ventricular hyprtrophy. Clin Biochem. (2007) 40:1020–5. doi: 10.1016/j.clinbiochem.2007.05.015 17604013

[13] Erbagci AB Araz M Erbagci A Tarakçioğlu M Namiduru ES. Serum prolidase activity as a marker of osteoporosis in type 2 diabetes mellitus. Clin Chem. (2002) 35:263–8. doi: 10.1016/S0009-9120(02)00305-3 12135686

[14] Altindag O Erel O Aksoy N Selek S Celik H Karaoglanoglu M. Inreased oxidative stres and its relation with collagen metabolism in knee osteoarthritis. Rheumatol Int. (2007) 27:339–44. doi: 10.1007/s00296-006-0247-8, PMID: 17096092

[15] Brosset B Myara I Fabre M Lemonnier A. Plasma prolidase and prolinase activity in alcoholic liver disease. Clin Chim Acta. (1988) 175:291–5. doi: 10.1016/0009-8981(88)90105-2, PMID: 3416488

[16] Evrenkaya TR Atasoyu EM Kara M Unver S Gultepe M. The role of prolidase activity in the diagnosis of uremic bone disease. Ren Fail. (2006) 28:271–4. doi: 10.1080/08860220600577726, PMID: 16771240

[17] Kaleli S Akkaya A Akdogan M Gültekin F. The effects of different treatments on prolidase and antioxidant enzyme activities in patients with bronchial asthma. Environ Toxicol Pharmacol. (2006) 22:35–9. doi: 10.1016/j.etap.2005.11.001, PMID: 21783683

[18] Reichert J Araujo AJ Goncalves CM Godoy I Chatkin JM Sales MP. Smoking cessation guidelines-2008. J Bras Pneumol. (2008) 34:845–80. doi: 10.1590/S1806-37132008001000014, PMID: 19009219

[19] Miller MR Hankinson J Brusasco V Burgos F Casaburi R Coates A. Standardisation of spirometry. Eur Respir J. (2005) 26:319–38. doi: 10.1183/09031936.05.00034805 16055882

[20] Isbilen E Kulaksizoglu S Kirmizioglu M Karuserci Komurcu O Tabur S. Role of prolidase activity and oxidative stress biomarkers in unexplained infertility. Int J Gynaecol Obstet. (2022) 156:430–5. doi: 10.1002/ijgo.13899, PMID: 34449881

[21] Pryor WA Stone K. Oxidants in cigarette smoke radicals, hydrogen peroxide, peroxynitrate and peroxynitrite. Ann N Y Acad Sci. (1993) 686:12–27. doi: 10.1111/j.1749-6632.1993.tb39148.x 8512242

[22] Ceylan E Aksoy N Gencer M Vural H Keles H Selek S. Evaluation of oxidative-antioxidative status and the L-arginine-nitic oxide pathway in asthmatic patients. Respir Med. (2005) 99:871–6. doi: 10.1016/j.rmed.2004.12.001, PMID: 15939249

[23] Gencer M Aksoy N Dagli EC Uzer E Aksoy S Selek S. Prolidase activity dysregulation and its correlation with oxidative-antioxidative status in chronic obstructive pulmonary disease. J Clin Lab Anal. (2011) 25:8–13. doi: 10.1002/jcla.20347, PMID: 21254236 PMC6647584

[24] Beck-Speier I Dayal N Karg E Maier KL Schumann G Schulz H. Oxidative stress and lipid mediators induced in alveolar macrophages by ultrafine particles. Free Radic Biol Med. (2005) 38:1080–92. doi: 10.1016/j.freeradbiomed.2005.01.004, PMID: 15780766

[25] Mahmood I Abdulla KS Othman SH. The total antioxidant status in cigarette smoking individuals. Med J Basrah Univ. (2007) 25:46–50. doi: 10.33762/mjbu.2007.48140

[26] Gilks CB Price K Wright JL Churg A. Antioxidant gene expression in rat lung after exposure to cigarette smoke. Am J Pathol. (1998) 152:269–78. PMID: 9422544 PMC1858137

[27] Zinellu E Zinellu A Fois AG Fois AS Piras B Carru C. Reliability and usefulness of different biomarkers of oxidative stress in chronic obstructive pulmonary disease. Oxidative Med Cell Longev. (2020) 2020:1–12. doi: 10.1155/2020/4982324, PMID: 32509143 PMC7244946

[28] Raitio A Tuommas H Kokkonen N Salo T Sorsa T Hanemaaijer R. Levels of matrix metalloproteinase-2,-9 and −8 in the skin, serum and saliva of smokers and non-smokers. Arch Dermatol Res. (2005) 297:242–8. doi: 10.1007/s00403-005-0597-1, PMID: 16215764

[29] Ekin S Arısoy A Gunbatar H Sertogullarindan B Sunnetcioglu A Sezen H. The relationships among the levels of oxidative and antioxidative parameters, FEV_1_ and prolidase activity in COPD. Redox Rep. (2017) 22:74–7. doi: 10.1080/13510002.2016.1139293, PMID: 26870880 PMC6837489

[30] Ergin Tuncay M Neselioglu S Asfuroglu Kalkan E Inan O Sena Akkus M Ates I. Modified proline metabolism and Prolidase enzyme in COVID-19. Lab Med. (2022) 53:453–8. doi: 10.1093/labmed/lmac017, PMID: 35394547 PMC9047239

[31] Uysal P Teksoz D Aksan H Durmus S Uslu-Besli L Cuhadaroglu C. Relationship between serum sialic acid levels and prolidase activity with airflow obstruction in patients with COPD. Medicine (Baltimore). (2022) 101:e28949. doi: 10.1097/MD.0000000000028949, PMID: 35356903 PMC10684178

[32] Türkbeyler I Demir T Pehlivan Y Kaplan DS Ceribasi AO Orkmez M. Prolidase could act as a diagnosis and treatment mediator in lung fibrosis. Inflammation. (2012) 35:1747–52. doi: 10.1007/s10753-012-9493-y, PMID: 22717888

[33] Gumus S Yaman H Ozcan O Deniz O Karaman B Cakir E. Serum prolidase activity in patients with pulmonary tuberculosis. Scand J Clin Lab Invest. (2011) 71:467–72. doi: 10.3109/00365513.2011.587021, PMID: 21722016

[34] Aslaner O. Comparison of oxidative effects of electronic cigarette and tobacco smoke exposure performed experimentally. Eur Addict Res. (2022) 28:41–7. doi: 10.1159/000518204 34515107

